# A high-performance brain–computer interface for finger decoding and quadcopter game control in an individual with paralysis

**DOI:** 10.1038/s41591-024-03341-8

**Published:** 2025-01-20

**Authors:** Matthew S. Willsey, Nishal P. Shah, Donald T. Avansino, Nick V. Hahn, Ryan M. Jamiolkowski, Foram B. Kamdar, Leigh R. Hochberg, Francis R. Willett, Jaimie M. Henderson

**Affiliations:** 1 Department of Neurosurgery, Stanford University, Stanford, CA USA; 2 Department of Neurosurgery, University of Michigan, Ann Arbor, MI USA; 3 Department of Biomedical Engineering, University of Michigan, Ann Arbor, MI USA; 4 Biointerfaces Institute, University of Michigan, Ann Arbor, MI USA; 5 Michigan Neuroscience Institute, University of Michigan, Ann Arbor, MI USA; 6 Howard Hughes Medical Institute at Stanford University, Stanford, CA USA; 7 Robert J. and Nancy D. Carney Institute for Brain Science, Brown University, Providence, RI USA; 8 School of Engineering, Brown University, Providence, RI USA; 9 VA RR&D Center for Neurorestoration and Neurotechnology, Rehabilitation R&D Service, VA Providence Healthcare System, Providence, RI USA; 10 Center for Neurotechnology and Neurorecovery, Department of Neurology, Massachusetts General Hospital, Harvard Medical School, Boston, MA USA; 11 Wu Tsai Neurosciences Institute, Stanford University, Stanford, CA USA; 12 Bio-X Institute, Stanford University, Stanford, CA USA

**Keywords:** Biomedical engineering, Neurological disorders

## Abstract

People with paralysis express unmet needs for peer support, leisure activities and sporting activities. Many within the general population rely on social media and massively multiplayer video games to address these needs. We developed a high-performance, finger-based brain–computer-interface system allowing continuous control of three independent finger groups, of which the thumb can be controlled in two dimensions, yielding a total of four degrees of freedom. The system was tested in a human research participant with tetraplegia due to spinal cord injury over sequential trials requiring fingers to reach and hold on targets, with an average acquisition rate of 76 targets per minute and completion time of 1.58 ± 0.06 seconds—comparing favorably to prior animal studies despite a twofold increase in the decoded degrees of freedom. More importantly, finger positions were then used to control a virtual quadcopter—the number-one restorative priority for the participant—using a brain-to-finger-to-computer interface to allow dexterous navigation around fixed- and random-ringed obstacle courses. The participant expressed or demonstrated a sense of enablement, recreation and social connectedness that addresses many of the unmet needs of people with paralysis.

## Abstract (web summary)

A finger-based brain–computer interface was developed for a person with tetraplegia to allow him to fly a virtual quadcopter, an innovation that can lead to improved social connectedness, recreation and a sense of enablement.

## Main

More than 5 million people in the United States live with severe motor impairments^1^. Although many basic needs of people with paralysis are being met, unmet needs for peer support, leisure activities and sports are reported, respectively, by 79%, 50% and 63% of surveyed people with paralysis from spinal cord injury^2^. People with motor impairments that spare enough function to manipulate a video game controller have turned to video games for social connectedness and a competitive outlet^3,4^. In a survey of players with and without disabilities^3^, a variety of themes emerged (for example, recreation, artistic expression, social connectedness); however, in those with disabilities, many expressed a theme of enablement, meaning both equality with able-bodied players and overcoming their disability. Even with assistive/adaptive technologies, gamers with motor impairments often have to play at an easier level of difficulty^5^ or avoid multiplayer games with able-bodied players^6^ that often require dexterous multieffector control^4,7^. Brain–computer interfaces (BCIs), increasingly recognized as a potential solution for motor restoration, could enable sophisticated control of video games for people with paralysis—and, more broadly, control of digital interfaces for social networking or remote work.

In motor BCIs, most effort has focused on controlling single effectors such as computer cursors for point-and-click cursor control and robotic arms for reaching and grasping (where fingers moved as a group)^8–16^. To expand object manipulation, ref. ^17^ continuously decoded linear combinations of four distinct grasping postures. Beyond simple grasps, providing reliable individuated finger control would allow activities such as typing, playing a musical instrument or manipulating a multieffector digital interface such as a video game controller. In humans, finger decoding has only been demonstrated in prediction in offline analyses or classification from recorded neural activity^18–21^. Continuous finger decoding has been limited to two finger groups (two degrees of freedom (DOF))^22,23^ in non-human primates (NHPs).

In a human research participant with paralysis, we developed a finger BCI system that is more functional than previous devices^23^ and is capable of continuously decoding three independent finger groups, of which the thumb was decoded in two dimensions, yielding a total of four DOF (doubling the decoded DOF in NHPs^22,23^). We used the decoded finger movements to provide independent digital endpoints for control of a virtual quadcopter, in a demonstration of a high-performance, continuous, finger-based intracortical BCI (iBCI)—illustrating the power of intuitive, individuated finger control as an intermediary representation between the mapping of brain recordings to computer interfaces. Just as able-bodied users of digital systems use their fingers to manipulate keyboards and game controllers, this system allows an intuitive framework for a brain-controlled digital interface, providing opportunities for recreation and socialization as well as eliciting feelings of enablement.

## Results

**Fig. 1:**
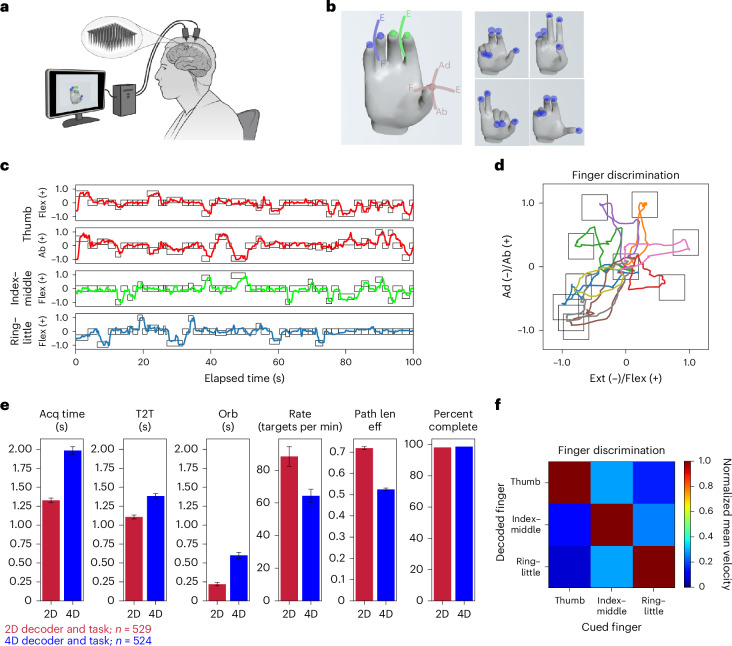
iBCI system for dexterous finger movements. **a**, A computer display is placed in front of the participant so that he can perform a finger task with a virtual hand. During closed-loop control, the electrical activity from the array is mapped to a control signal for the virtual fingers. Panel adapted from ref. ^27^. **b**, Left, thumb moves in two dimensions, abduction (Ab) and adduction (Ad) (flexion/extension and abduction/adduction), and index–middle and ring–little move in a 1D arc. Right, trials showing typical targets for all three finger groups for the four-DOF task. **c**, A 100-s time segment of typical decoded movements are depicted for the three-finger group, four-DOF task. Trajectories are described along a range of −1 to 1, where 1 denotes full flexion (flex) or abduction (ab) and −1 denotes extension (ext) or adduction (ad). **d**, The trajectories for an illustrative 50-trial block of 2D thumb movements, showing only trials where the thumb travels a distance greater than 0.3. Each color represents distinct paired, center-out-center trial. **e**, Summary statistics comparing the two- and four-DOF tasks for acquisition time (Acq time), time to target (T2T), orbiting time (Orb), acquisition rate (Rate), path length efficiency (Path len eff) and the percent of trials successfully completed (Percent complete). The error bars represent the standard error of the mean. There were *n* = 529 trials and *n* = 524 trials for the two- and four-DOF tasks, respectively. **f**, Four blocks in which only one finger was cued per trial, illustrating individuated control of fingers. The mean velocity per trial was calculated during the ‘Go’ period for each finger and normalized by the mean value of the finger group with the highest mean value. F, flexion; E, extension.

**Extended Data Fig. 1:**
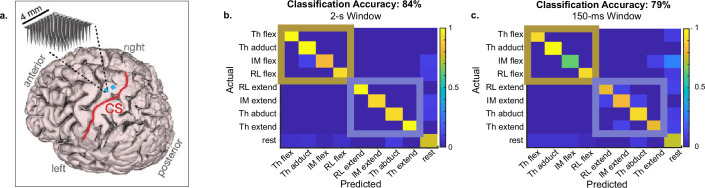
The intracortical brain-computer interface system for dexterous finger movements. **a**, MRI reconstruction of the participant’s brain with the implant locations depicted as blue squares. Two 96-channel silicon microelectrode arrays were placed in hand ‘knob’ area of left precentral gyrus in 2016. The red line indicates the central sulcus (CS). Panel from Deo et al.^41^. **b** and **c**, Confusion matrices showing the probability of correctly classifying attempted finger movements using 2 s, **b**, and 150-ms, **c**, windows from an offline analysis on open-loop data from day 2423. IM, index-middle; RL, ring-little; TH, thumb.

Multiunit neural activity was recorded from two 96-channel silicon microelectrode arrays placed in the hand ‘knob’ area of the left precentral gyrus in one participant (‘T5’) enrolled in the BrainGate2 pilot clinical trial (Extended Data Fig. 1a). T5 was a 69-year-old right-handed man with C4 AIS C spinal cord injury, leaving only non-functional twitches and micromotion of his upper and lower extremities. A virtual hand was displayed to the participant using Unity (v.2021.3.9f1, Unity Technologies), as shown in Fig. 1a. The thumb was designed to move along a two-dimensional (2D) surface defined by the flexion–extension and abduction–adduction axes (Fig. 1b). Both the index–middle and ring–little fingers moved as separate groups in a one-dimensional (1D) arc constrained to the flexion–extension axis. Tuning of the microelectrode arrays to finger movements was confirmed (Extended Data Fig. 1b,c).

### Closed-loop real-time control of a two- and four-DOF finger task

**Extended Data Fig. 2:**
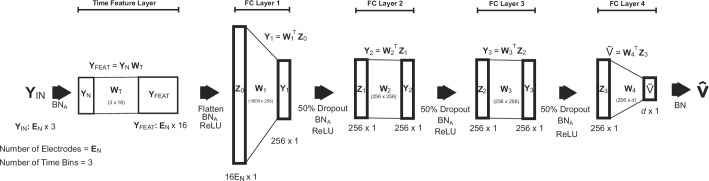
Decoding algorithm. The input ***Y***_*IN*_ is *E*_*N*_ x 3 input matrix, where *E*_*N*_ is the number of electrodes (192) and 3 represents the most recent 3 50-ms bins. The output variable, $\hat{{\boldsymbol{v}}}$, represents a normalized vector of each of *d* finger velocities. The actual decoded velocities were calculated by applying an empirically calculated mean value and gain value. Linear layers (*W*_*T*_, *W*_*1*_-*W*_*3*_) included a learnable bias term except for the final linear layer, *W*_*4*_, to reduce the magnitude of non-zero means. All instances of batchnorm, BN_A_, were implemented with affine = *True* except for the final batchnorm, BN, where affine = *False* in an attempt to reduce the reliance of the decoding algorithm on an offset correction from the final batchnorm block. During training mode batchnorm layer, BN, did not correct for non-zero means or apply a mean correction to force the final linear layer, *W*_*4*_, to learn an output signal with zero mean. BN, batchnorm; FC, fully connected; ReLU, rectified linear unit. Figure adapted from (Willsey et al. 2022)^23^.

To perform closed-loop continuous decoding, a temporally convolved feed-forward neural network, adapted from ref. ^23^, mapped spike-band power (SBP)^24^ to finger velocities used to control virtual finger movements on screen (Extended Data Fig. 2). The network parameters were initially trained from open-loop trials where the participant attempted to move his fingers in sync with moving fingers on the hand avatar. The decoding algorithm was then used in closed-loop training trials; the parameters are refined by assuming that decoded movements away from intended targets were errors.

**Extended Data Fig. 3:**
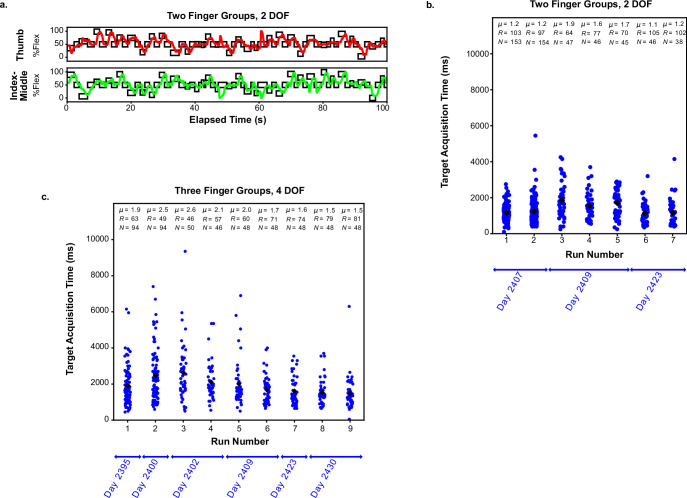
Closed-loop decoding for a 2- and 4-degree-of-freedom (DOF) finger task. **a**, A 100-s time segment of typical decoded movements is depicted for the 2-finger group, 2-DOF task. Trajectories are described as a percentage of flexion (%Flex). Distributions of target acquisition times (ms) for the 2-DOF task, **b**, and 4-DOF task, **c**, over multiple blocks of each task. Each dot corresponds to a trial, and the black diamond indicates the mean value. *N* denotes number of trials per block, R denotes the rate of targets acquired in targets/min, and *µ* denotes the mean value.

Two sets of tasks were performed. To translate findings from earlier NHP studies^22,23^, we sought to demonstrate decoding of two finger groups (2D task) in our human research participant (in this task, the thumb was constrained only to the flexion–extension axis). T5 was cued to move both the thumb and index–middle groups from a center position to a random target within the active range of motion of the fingers. On the subsequent trial, targets were placed back at center. To successfully complete a trial, the fingers had to hold on the targets for 500 ms, and 10 s were allowed to complete the trial (sample trajectories in Extended Data Fig. 3a; see Supplementary Video 1).

To expand on the functionality demonstrated in NHP studies, task complexity was increased by introducing a 4D task with 2D thumb movements and 1D movements of the index–middle group and the ring–little group (Fig. 1c). On each trial, two finger groups were randomly selected for new targets while the target for the third finger group remained in the same position as the previous trial, and movements of all fingers were continuously and simultaneously decoded and controlled. Typical target trajectories for this expanded 4D task are shown in Fig. 1c, and 2D trajectories of the thumb movements are illustrated in Fig. 1d. Supplementary Video 2 depicts this task.

**Extended Data Fig. 4:**
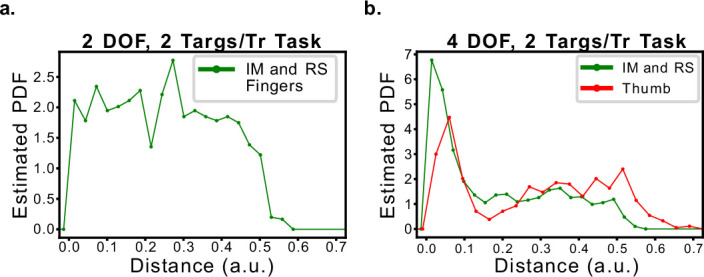
Distribution of finger distances. For the 2D and 4D task with 2 new targets/trial (in Fig. 1e), the distribution of distances for successful trials for the **(a)** 2D decoder and **(b)** 4D decoder. The histogram is normalized so that the area under the curve equals 1. In **a**, the green curve represents the distance for index-middle (IM) finger and ring-little (RL) finger flexion/extension combined over all trials for the 2D decoder and task trials in Fig. 1e. In **b**, the green curve represents combined IM finger and RL finger flexion/extension distances, and the orange curve represents the 2D distance for the thumb (combining the 2D components of flexion/extension and abduction/adduction) for trials using the 4D decoder and task in Fig. 1e. a.u., arbitrary units; DOF, degree of freedom; PDF, probability distribution function.

The closed-loop decoding performance for the 2D and 4D decoder was compared using 529 trials (3 days) for the 2D decoder and 524 trials (6 days) for the 4D decoder (Fig. 1e). For the 2D decoder, the mean acquisition time was 1.33 ± 0.03 s, the target acquisition rate was 88 ± 6 targets per minute, and 98.1% of trials were successfully completed. For the 4D decoder, the mean acquisition time was 1.98 ± 0.05 s, the target acquisition rate was 64 ± 4 targets per minute, and 98.7% of trials were successfully completed. The acquisition times for each trial (population data) is shown graphically in Extended Data Fig. 3b for the 2D decoder and Extended Data Fig. 3c for the 4D decoder. Typical finger distances per trial are shown graphically in Extended Data Fig. 4a for the 2D task and Extended Data Fig. 4b for the 4D task.

In comparison to the 2D decoder and task, the acquisition times were increased by 50% for the 4D decoder and task (*P* < 10^−10^, *t* = 11.00, d.f. = 1,051, confidence interval (CI) = 540 to 775 ms), and factors such as increased demands on decoding accuracy and the difficulty in keeping more fingers stationary on the targets (that is, signal-independent noise) may have led to slower performance. However, after the participant grew more accustomed to the task (final four blocks), acquisition time for the 4D decoder dropped by an average of 0.4 s to 1.58 ± 0.06 s (a target acquisition rate of 76 ± 2 targets per minute), and 100% of trials were completed. To compare this work with the previous NHP two-finger task where throughput varied from 1.98 to 3.04 bps with a variety of decoding algorithms^23,25^, throughput for the current method was calculated as 2.60 ± 0.12 bps (see Methods for details). Table 1 summarizes statistics for the 4D decoder/task and 2D decoder/task.

**Table 1:** Performance metrics for 2D and 4D finger decoding

	2D decoder	4D decoder	4D decoder (last four blocks)
Number of trials	529	524	192
Number of days	3	6	3
Acquisition time (ms)	1,330 ± 30	1,980 ± 50	1,580 ± 60
Time to target (ms)	1,110 ± 30	1,380 ± 30	1,200 ± 40
Orbiting time (ms)	220 ± 20	600 ± 40	380 ± 50
Targets per minute	88 ± 6	64 ± 4	76 ± 2
Percent completed	98.1%	98.7%	100%
Path length	0.718 ± 0.007	0.524 ± 0.007	0.580 ± 0.010

Statistical data are reported as mean ± s.e.m.

Finger discrimination during closed-loop control was demonstrated on a 4D task cuing one finger group per trial. The mean velocity of the finger groups was calculated during the ‘Go’ period. The movement of the non-cued fingers was substantially less than the movement of the cued finger (Fig. 1f), demonstrating finger individuation.

**Extended Data Fig. 5:**
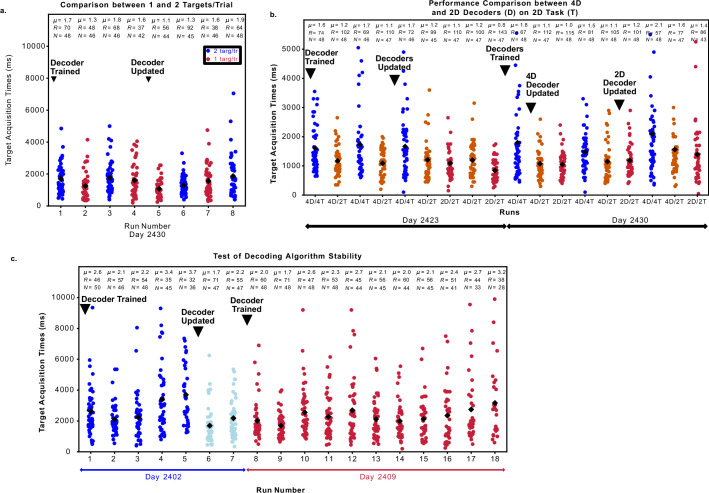
Target acquisition times for a variety of comparisons. **a**, Performance comparison for 1 vs. 2 new targets/trial (targ/tr). Population data for the target acquisition times (ms) using the 4D decoder for 2 new targets/trial (blue) and 1 new target/trial (red) over blocks of the task. Each dot corresponds to a trial, and the black diamond indicates the mean value. *N* denotes the number of trials per block, *µ* denotes the mean value, and R denotes the rate of targets acquired in targets/min. **b**, The 4D and 2D decoders on 2D task. Target acquisition times (ms) for the 4D and 2D decoders are compared on the 2D task with 2 new targets/trial task (2T). The 4D decoder is also run on the 3-finger group, 4D task (4T) with 2 new targets/trial. The blocks on the 2423 and 2430 trial days of data collection represent consecutive blocks without re-training unless otherwise indicated. The labels 4D/4T indicate the 4D decoder run on 4T; 4D/2T indicates the 4D decoder on 2T; and 2D/2T indicates the 2D decoder on 2T. **c**, Decoder stability test for 2 trial days using the 4-DOF decoder for 2 new targets/trial. For days 2402 (blue and light blue) and 2409 (red), the decoder was trained (upside down triangle with “Decoder Trained”) and then used in consecutive blocks until trials could not be reliably completed. On day 2402, the decoder was re-trained (“Decoder Updated”) to demonstrate the recovery of performance on 2 subsequent blocks (light blue).

The 4D decoder was compared for tasks with one (178 trials) and two cued finger-group movements (187 trials). The mean acquisition time was 1.37 ± 0.06 s for one finger and 1.66 ± 0.07 s for two fingers (*P* = 0.0036, *t* = −2.93, d.f. = 363, CI = −485 to −96 ms). The target acquisition rate was 45 ± 4 targets per minute for one finger and 74 ± 6 targets per minute for two cued fingers (*P* = 0.0092, *t* = 3.78, d.f. = 6, CI = 10 to 48 targets per minute) (Supplementary Video 3, Extended Data Fig. 5a and Extended Data Table 1).

### Dimensionality of the neural activity

**Fig. 2:**
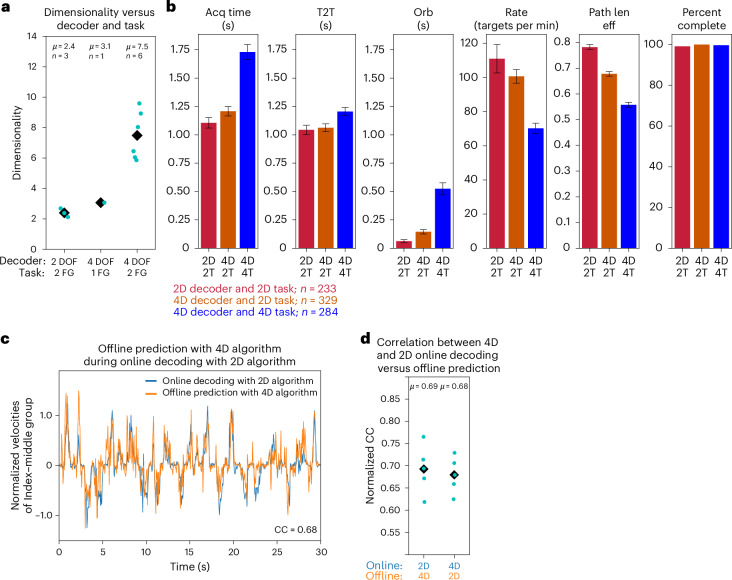
Dimensionality. **a**, Dimensionality of the neural activity during closed-loop decoding using either the 2D decoder/task or the 4D decoder/task for either one or two new finger-group targets (1 FG or 2 FG) per trial. Light blue dots represent data from a day, and black diamonds represent the mean, *µ*. **b**, Summary statistics comparing the 2D decoder on the 2D task (2D, 2T; *n* = 233 trials), the 4D decoder on the 2D task (4D, 2T; *n* = 329 trials) and the 4D decoder on the 4D task (4D, 4T; *n* = 284 trials) based on the acquisition time, time to target, orbiting time, acquisition rate, path length efficiency and the percent of trials successfully completed. The error bars represent the standard error of the mean. **c**, A typical online block showing the decoded index–middle finger group velocities using the 2D on 2T (blue) during an online block. Offline, the 4D decoding algorithm was used to predict index–middle group velocities from the same block (orange). The normalized CC between the online and offline signals is given in the bottom-right corner. Units are denoted so that the range of motion for each DOF is unity. **d**, For the ten blocks on the 2D task, the 4D decoding algorithm was used to predict finger velocities during online blocks using the 2D decoder (online 2D in blue, offline 4D in orange), and the 2D decoding algorithm was used to predict online velocities using the 4D decoder (online 4D in blue, offline 2D in orange). CC between the offline and online signals is represented by dots and averaged across both finger groups. The diamond and *µ* denote the mean value for the average of all five blocks for each paired comparison.

With a potential nonlinear relationship between neural activity and finger movement^22,26^, the dimensionality of the neural activity might be nonlinearly related to increases in decoded DOF. The dimensionality of the neural data during 4D and 2D decoding was calculated using the participation ratio used in ref. ^27^. The average dimensionality of neural activity was 2.4 for the 2D decoder, 3.1 for the 4D decoder with one new target/trial and 7.5 for the 4D decoder with two new targets/trial (Fig. 2a). If the dimensionality of the neural activity varied linearly with the decoded DOF, the dimensionality of the 4D decoder would be twice that of the 2D decoder: that is, 2 × 2.4 = 4.8; however, dimensionality using the 4D decoder was found to be 7.5, 56% more than the expected value of 4.8 (*P* = 0.028, *t* = 2.77, d.f. = 7, CI = 0.39 to 4.99). Thus, the dimensionality of combined finger movements was greater than the sum of the individual components. Although the dimensionality of combined movements awaits further study, these results may imply that some neurons encode both single and/or combined movements, as suggested by studies showing single units can encode the muscle and whether the muscle functions as agonist or antagonist^28^.

### Effect of number of active DOF on decoding

Despite increased dimensionality in neural activity when decoding more DOF, it is unclear whether decoding more DOF impacts the mapping of the neural activity when decoding a lower number of DOF. The neural representation of the DOF decoded in the 2D task (thumb and index–middle flexion/extension) could change during the 4D task, for example, if a different control strategy is required for the 4D compared to the 2D task—similar to how new control strategies can be developed to account for a perturbation in the mapping from neural activity to the DOF^29^. Alternatively, the original neural representation could be suppressed when tasked with decoding additional fingers, as is the case when decoding unilateral versus bilateral movements^30^. A third competing hypothesis is that the neural representation of finger movement in the 2D task is preserved in the 4D task, similar to preservation of neural representation between open-loop motor imagery and closed-loop control^31^.

To explore these hypotheses, 2D and 4D decoders were trained and compared by testing on the two shared DOF (thumb flexion/extension and index–middle group flexion/extension) over two days (662 trials), in alternating trials (Fig. 2b and Extended Data Table 2). The mean acquisition time was 1.11 ± 0.05 s for the 2D decoder on the 2D task (*n* = 233), 1.73 ± 0.07 s for the 4D decoder on the 4D task (*n* = 284) and 1.21 ± 0.04 s for the 4D decoder on the 2D task (*n* = 329). Supplementary Video 4 displays the 4D decoder on the 2D task. The trial-by-trial acquisition times for this comparison are given in Extended Data Fig. 5b. The 4D decoder performed much closer to the 2D decoder when restricted to the same 2D task (9.2% increased acquisition times, *P* = 0.10, *t* = 1.64, d.f. = 560, CI = −20 to 224 ms). Thus, training a decoder on an expanded set of movements does not appear to substantially degrade decoding performance (summarized in Fig. 2b and Extended Data Table 2).

The mapping from neural activity to the original two DOF was compared for both 2D and 4D decoders. To do this, the 4D decoder was used to predict the velocities decoded by the 2D decoder on the 2D task and vice versa. The predicted velocities from the 4D decoder were similar to those decoded in online blocks by the 2D decoder (Fig. 2c). To quantify this comparison, the normalized cross-correlation (CC) function was calculated between the decoded and predicted velocities during the eight blocks (Fig. 2d). The results were separated based on whether the online decoded velocities were from the 4D or 2D decoders. The CC when the 4D algorithm predicted the 2D decoded velocities was 0.69 ± 0.02, and when the 2D algorithm predicted the 4D decoded velocities, the CC was 0.68 ± 0.02 (Fig. 2c). Thus, the neural representation of finger movements appeared similar despite actively controlling more DOF, consistent with other reports where many DOF are represented within the same neural population in motor cortex^17,30^.

### Dependency of decoding accuracy on channel count

**Fig. 3:**
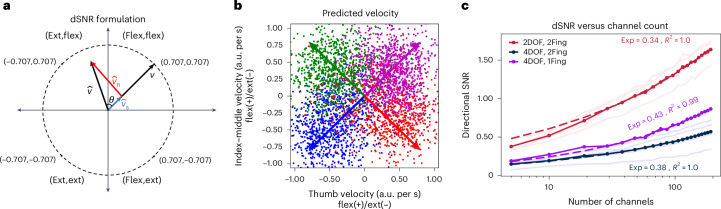
SNR ratio versus channel count. **a**, Graphical representation for vectorized dSNR for the 2D decoder and task. The positive *x* axis represents velocities flexing the thumb, and negative values represent velocities extending the thumb. The *y* axis represents velocities flexing the index–middle finger group when positive and extending the finger group when negative. Because the signal vector, **v**, is assumed to be a normalized target vector, the values of **v** can be only the four points indicated on the circle. The decoded/predicted velocity, $\boldsymbol{\hat{{v}}}$, will lie at an angle *θ* to **v** and can be decomposed into a parallel signal component, ${\boldsymbol{\hat{{v}}}}_{\bf{s}}$ and a perpendicular noise component, ${\boldsymbol{\hat{{v}}}}_{\bf{n}}$. These components can be used to calculate dSNR. **b**, Velocities predicted by linear regression (using all *N*_c_ = 192 channels) that maps neural activity to finger velocities, which together with the intended finger movements are used to calculate dSNR. The arrows represent the ideal/truth value for each possible finger position based on the assumed intended finger movement. **c**, The dSNR as a function of channel count for the 2D decoder on the two-target/trial task (2Fing, red), 4D decoder on the two-target/trial task (2Fing, blue) and 4D decoder on the one target/trial task (1Fing, purple). An empirical calculation of dSNR for each day is depicted in lightly colored lines and the mean value as the dark solid line. The dashed lines correspond to a linear, least-squares fit for the log–log relationship in equation (1), where *m* denotes the log–log slope and *R*^2^ denotes the coefficient of determination of the linear fit. a.u., arbitrary units; Fing, finger.

Because newer BCI devices will have more electrodes than used herein, we explored whether increased channels would be expected to increase decoding accuracy with a vector-based, sample-by-sample signal-to-noise ratio (SNR) metric, directional SNR (dSNR; Fig. 3a). The predicted/decoded finger velocities were compared with idealized velocities inferred from intended finger movements. The component of the predicted/decoded velocities consistent with idealized velocities (that is, the component parallel to the idealized vector of finger velocities) was considered the signal component, whereas the predicted/decoded velocities inconsistent (that is, the component orthogonal to the idealized velocity vector) were considered noise. dSNR was the ratio of the expected signal mean over the square root of the noise power.

The value of dSNR was calculated during a ‘Go’ period of closed-loop trials (defined as 200–700 ms from trial onset) of two- and three-finger decoding (Table 2). On each day, linear regression was used to train a mapping (against the intended finger direction) to convert SBP to finger velocities (using sixfold cross-validation). Predicted velocities (calculated from all 192 input channels) grouped along the idealized, intended directions (Fig. 3b).

**Table 2:** Data sessions and training the closed-loop decoding algorithm

S no.	Day	Task	D no.	DOF	OL blocks (trials)	CL blocks (trials)^a^	CL training time (min)	Figure	Notes
1	2395	CL finger decoding	1	4	2 (200)	7 (467)	24.3	1, 3, ED 3, ED 4	
2	2400	CL finger decoding	2	4	2 (200)	3 (180)	9.8	1, 3, ED 3, ED 4	
3	2402	CL finger decoding	3	4	2 (200)	5 (252)	14.1	1, 3, ED 3–5	
			3b			+5 (250)	16.3	ED 5	
4	2407	CL finger decoding	4	2	2 (200)	2 (100)	5.9	1, 3, ED 3, ED 4	
5	2409	CL finger decoding	5	4	2 (200)	9 (450)	25.7	1, 3, ED 3–5	
			6	2	2 (200)	2 (100)	11.1	1, 3, ED 3,ED 4	
6	2423	CL finger decoding	7	4	2 (200)	12 (585)	35.8	1–3, ED 1, ED 3–5	Open-loop data were used for the confusion plots in Extended Data Fig. 1. Closed-loop data were used in Figs. 2–4.
			7b			+2 (100)	5.5	2, ED 5	
			8	2	2 (200)	7 (297)	20.2	1, ED 3, ED 4	Attempted to overtrain the decoder by ReFIT training it over many closed-loop blocks after 100% of trials were completed
			8b			+3 (150)	5.1	2, 3, ED 5	Includes 1 block (50 trials) using the 4D decoder
7	2430	CL finger decoding	9	4	2 (200)	3 (150)	8.2	1, ED 3, ED 4	
			9b			+2 (100)	5.1	2, ED 5	
			9c			+1 (50)	2.5	2, ED 5	
			9d			+7 (331)	16.8	1–3ED 5	Used in Fig. 3 for more trials (than earlier blocks)
			9e			+1 (50)	2.0	1–3, ED 5	Used in Fig. 3 for more trials (than earlier blocks)
			10	2	1 (100)	1 (50)	1.5	2, 3, ED 5	Used in Fig. 3 for more trials (than earlier blocks)
			10b			+1 (50)	1.5	2, 3,ED 5	Used in Fig. 3 for more trials (than earlier blocks)
			10c			+1 (50)	2.0	2a, 3	Used in Fig. 3 for more trials (than earlier blocks)
8	2520	QC obstacle course	11	4	2 (200)	3 (150)	8.3		
			11b			+4 (200)	10.3	4	
			11c			+2 (100)	5.8		
			11d			+2 (100)	4.7		
9	2569	QC random rings	12	4	2 (200)	6 (269)	20.4		Open-loop blocks were performed using common average referencing, and closed-loop blocks used linear regression referencing

CL, closed-loop; CL training time, time to complete the closed-loop training blocks; D no., decoder number; ED, Extended Data Fig.; OL, open-loop; OL blocks required 5 min per 100 trials; QC, quadcopter; S, session.

^a^+, additional training blocks and trials.

To determine the dependency dSNR on channel count, a linear mapping of SBP to velocities was trained for a given number of *N* channels, which was used to calculate dSNR (using sixfold cross-validation and where dSNR was the average using 25 sets of *N* randomly selected channels; see Methods). For both the 2D and 4D tasks requiring movement of two simultaneous finger groups, dSNR did not saturate with increasing numbers of input channels (Fig. 3c). Because the dSNR metric assumes that both finger groups are simultaneously moving toward their respective targets (as opposed to moving one at a time), the simpler 4D task that required only one cued finger movement/trial was also used (Fig. 3c). Using the dSNR data for the highest 75% of channel counts of each curve, a log–log relationship between channel count and dSNR was empirically fit to a linear relationship. The empirical fit of the log–log relationship was strongly linear, with a coefficient of determination, *R*^2^, between 0.99 and 1.00 and a slope, *m*, of 0.34 for the 2D task moving two fingers, 0.38 for the 4D task moving two fingers and 0.43 for the behaviorally simpler 4D task moving one finger. Given the high *R*^2^ value, the empirical relationship between the dSNR and channel count fit the relationship in equation (1):

1 $$\rm{dSNR}=B\times{\it{N}}_{\rm{C}}^{\it{m}}$$

where B is an arbitrary constant, *m* is the slope (varying 0.34–0.43) and *N*_C_ is the channel count. The empirically determined growth (*m* = 0.34 to 0.43) could be less than the ideal of *m* = 0.5 because of behavioral confounders or violations of noise assumptions (independent, identically distributed gaussian noise; Methods).

### Translation of a finger iBCI to virtual quadcopter control

**Fig. 4:**
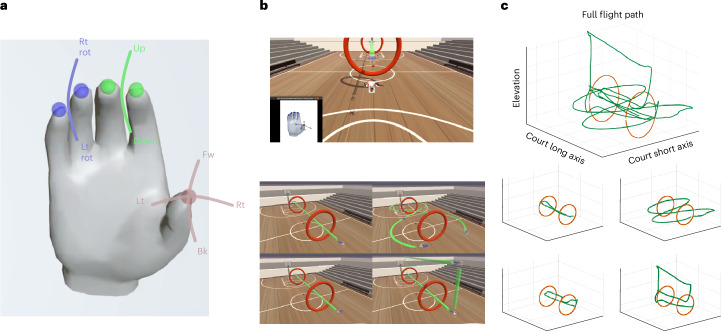
Finger iBCI translated to virtual quadcopter control. **a**, Mapping finger position to quadcopter velocities. The thumb position is mapped to forward (Fw), backward (Bk), left (Lt) and right (Rt) translation velocity. The index–middle finger group position is mapped to velocities directed up and down in elevation. The position of the ring–little finger group is mapped to right rotation (Rt rot) and left rotation (Lt rot) velocities. **b**, Top, the layout for quadcopter control showing the virtual quadcopter in the center of the screen. A visualization of the hand indicating the neutral points for the finger groups and cardinal directions of the thumb is also visible. The rings are seen in the center of the display, and the green straight line indicates the trajectory the quadcopter is to follow along the obstacle course. Bottom, the quadcopter obstacle course demonstrates the four-DOF control required to complete the 4.5-lap obstacle course. The top-left path requires the quadcopter to move forward, turn around and move forward through the same rings to return to the starting point (one lap). The top-right path requires the participant to simultaneously move forward and turn to complete two ‘figure-8’ paths around the rings and back to the starting point (one lap). The bottom-left path requires him to move left through both rings, stop and then move right back through the rings (one lap). The bottom-right path requires moving forward through the rings, increasing the elevation, moving backward over top of the rings, decreasing elevation and then moving forward through both rings to the ending point (1.5 laps). **c**, Top, an exemplary full-flight path during a block of the obstacle course. Bottom, the flight path is separated into laps corresponding to the planned flight path for each lap in **b** (bottom).

Although an obvious clinical application of a finger iBCI is to restore fine motor control for a robotic arm^9^ or to reanimate the native limb^10^, a finger iBCI system could also be an intuitive approach to controlling multiple simultaneous digital endpoints, extending the functionality of 2D cursor control^14^. Another application for multiple-DOF finger control is video gaming, aimed at enabling people with disabilities to participate with others. To this end, each finger movement was mapped to a DOF for control of a virtual quadcopter (Fig. 4a). Unlike a previous implementation of a flight simulator^32^, the finger positions were mapped directly to velocity control of the quadcopter and not transformed into ‘quadcopter space’ during retraining. Mapping finger positions to velocity control could also allow a general-purpose control paradigm for a variety of games. The only task-specific adaptation was to apply a low-level velocity back to neutral when the fingers were within 10% (of the total range of motion) of the neutral position. This kept the fingers in the neutral position unless the participant deliberately moved them. The positions of the fingers were visible in the bottom-left portion of the screen with annotations indicating the neutral position of each finger and the cardinal directions for the thumb movements (Fig. 4b, top).

To demonstrate all the possible four-DOF movements, an obstacle course was created (Fig. 4b, bottom) where each course segment could demonstrate at least one of the movements. On a single day of testing, the participant controlled the quadcopter over the complete obstacle course 12 times with an average block time of 222 s and a standard deviation of 45 s. An exemplary block, completed in 163 s, is shown in Supplementary Video 5 with the flight path depicted in Fig. 4c. Because all fingers could be simultaneously decoded, multiple quadcopter movements could be combined with multiple finger movements, such as when the quadcopter moves forward and turns during the figure-8 segment of the obstacle course. Furthermore, because the finger positions lie along a continuum, a range of velocities can be provided for quadcopter control, which allows for high-velocity movements to cover large distances or low-velocity movements for fine adjustments.

Although the obstacle course demonstrates four-DOF control, the quadcopter was also tested in a less scripted, free-form task in which the participant was instructed to fly the quadcopter through randomly appearing rings (timeout every 20 s). This task illustrates reaction time, corrective maneuverability and the ability to combine simultaneous DOF. After training the decoder, the participant was asked to fly through the rings. Over 10 min, he flew through 28 rings (2.8 rings per minute); an illustrative segment from this session is given in Supplementary Video 6. Importantly, performance was impacted not only by decoding accuracy but also largely by behavioral factors, as even able-bodied operators using a unimanual quadcopter control might find the task challenging.

### User experience

As seen in other non-BCI studies that illustrate the clinical impact of video games in people with disabilities^3^, T5 expressed themes of social connectedness, enablement and recreation during BCI control of the quadcopter. He expressed on multiple occasions (even before enrollment in the clinical trial) that one of his most important personal priorities was to use a BCI to control a quadcopter; he felt controlling a quadcopter would enable him, for the first time since his injury, to figuratively “rise up” from his bed/chair. He looked forward to the quadcopter sessions, frequently asked when the next quadcopter session was and often requested that we send video clips of quadcopter flights to his friend. When not running protocoled experiments, he enjoyed spontaneously flying the quadcopter. He would constantly ask for more “stick time” so he could improve his performance and exclaimed once that “I feel like we can work until 9 tonight”. Fatigue did not appear to be a factor in quadcopter control, with T5 never requesting to terminate or shorten any of the nine sessions included in this study. Ultimately, this work was the culmination of a long-held goal seen by both the research team and the participant as a joint collaborative achievement.

T5 provided substantial feedback on using the system. He felt that training was “not tedious” and that training on a random finger task was “better training” and “a lot more what the [quadcopter] is like” than a center-out task. Conversations led to a visual display of the fingers (with crosshairs to denote the neutral position) during closed-loop, real-time quadcopter control. He reported the finger graphic initially required his attention a couple of times a second, although he felt control of the drone without looking at the fingers was feasible and learnable. With practice, he referenced the fingers once every few seconds saying, “when the drone is moving and the fingers are moving, it’s easier and faster to just look at the drone”.

T5 described the intuitive control: “It’s like riding your bicycle on your way to work, ‘what am I going to do at work today’, and you’re still shifting gears on your bike and moving right along”. T5 emphasized the dexterity required, saying “Flying it is tiny little finesses off a middle line, a little bit up, a little bit down”, and compared the control to playing a delicate musical instrument. When transitioning from training the fingers to controlling the quadcopter, he explained: “It’s like if you’re a clarinet player and you pick up someone else’s clarinet. You know the difference instantly and there is a little learning curve involved but that’s based on you have an implied competency with your clarinet”. He described the context difference between the fingers and quadcopter control explaining that the quadcopter control was “more sensitive than fingers” and he just had “to tickle it a direction”. He also emphasized the importance of individualization of the fingers and how failure of individualization degrades performance: “when you pull down with your [little finger], the other two finger [groups] are supposed to just stay there… but they track with the [little finger], which is what throws me off and the whole thing goes down and to the left instead of just left or whatever it is”.

## Discussion

People with paralysis often express unmet needs for peer support, leisure activities and sports^2^. Many people with motor impairments have turned to video games for social connectedness and a competitive outlet^3,4^ and have expressed a sense of enablement over their disability^3^. We developed the most capable finger BCI to date that enables continuous control of three highly individuated finger groups, of which the thumb moved in two dimensions (acquiring more than 75 targets per minute). Like how able-bodied users manipulate video game controllers with their fingers, we introduce an intuitive, finger-based iBCI where decoded finger positions controlled three digital effectors (with one effector moving in two dimensions) for high-performance, four-DOF control of a virtual quadcopter. The participant’s intuitive control was anecdotally described as being like playing a musical instrument and evoked a strong sense of enablement, recreation and socialization.

When people without motor deficits interface with computers or video games, the control paradigm is heavily reliant upon—or even requires—dexterous finger movements for a computer keyboard or video game controller to manipulate not just one cursor but multiple digital cursors, endpoints and buttons. However, most past research and commercial development has focused on using BCIs for 2D point/click cursor control^8,14,33,34^, which was used previously to control a quadcopter^35^ or flight simulator^32^ with constant thrust. Kryger et al.^32^ adapted two DOF from a previously developed prosthetic limb^17^ to control a flight simulator, reported to fly through 10 of 11 rings stacked in close sequence, with videos showing a potential need for more stability over a nearly linear flight path^32^. LaFleur et al.^35^ used an electroencephalographic-controlled quadcopter to navigate through 3.1 rings in 4 min (versus 12.0 in able-bodied controls using a keyboard). In contrast, our system allowed navigation through or around 18 rings—at peak performance—in less than 3 min, a more than sixfold increase in performance. The system was also capable of spontaneous free-form flight through randomly appearing rings. This approach to use fine motor control for iBCI-controlled video games can meet unmet needs of people with paralysis, as the entertainment value of video games is self-evident; 77% of video gamers were estimated to play socially in 2021 (ref. ^36^), and participation in multiplayer video games has been linked to social well-being and connectedness^3,4,37^, provides a competitive outlet^4^ and fosters teamwork^38^. These were precisely the themes communicated to us by our participant.

Increasing the channel count of the recording system could lead to increased decoding accuracy. Our surrogate for decoding accuracy, dSNR, did not appear to saturate at our current channel count of 192. A similar observation was made using iBCIs for speech decoding^39^, where a projected word error rate decrease from 23.8% to 8.4% was projected moving from a 128- to 256-electrode system, which was realized when a 2.5–9.8% word error rate was achieved with 256 electrodes^40^.

## Methods

### Clinical trial and participant

All research was performed while following all relevant regulations, and the participant underwent informed consent. The participant, T5, was enrolled as a participant in the BrainGate2 Neural Interface System clinical trial (NCT00912041, registered 3 June 2009) with an IDE from the FDA (IDE no. G090003). This study was approved by the Institutional Review Board of Stanford University (protocol no. 20804) and the Mass General Brigham Institutional Review Board (protocol no. 2009P000505). This investigation was pursued as part of the secondary outcome measure of the clinical trial. Participants were not compensated for participation. No sex/gender analysis was conducted, although participants were recruited regardless of sex/gender.

The participant, T5, was a 69-year-old right-handed man with C4 AIS C spinal cord injury, leaving only non-functional twitches and micromotion of his upper and lower extremities. In 2016, two 96-channel microelectrode arrays (Neuroport arrays with 1.5 mm electrode length; Blackrock Microsystems) were placed in the anatomically identified hand ‘knob’ area of the left precentral gyrus. Detailed array locations are depicted on an MRI-reconstructed graphic in Extended Data Fig. 1a (from ref. ^41^).

### Participant sessions

A total of nine sessions of 2–5 h per session between trial days 2395 and 2569 were used to demonstrate online, closed-loop finger decoding and quadcopter control. The participant lay flat in bed with the monitor positioned above and slightly to his left so that he could keep his neck in the neutral position. Data were collected in roughly 1–10 min blocks. In between blocks, T5 was encouraged to rest as desired. Descriptions of the data collection sessions are shown in Table 2.

### Finger tasks

A virtual finger display was developed in Unity (v.2021.3.9f1) that allows control of virtual fingers. The thumb was programmed to allow movement in two dimensions (flexion/extension and abduction/adduction), the index–middle fingers were grouped to move together within a 1D flexion/extension arc, and the ring–little fingers were grouped together to move in a 1D flexion/extension arc. By supplying a value between 0 and 1 for each of the four DOF, the finger position could be placed at continuously varying positions between full flexion and extension or abduction and adduction. Finger position values were set to follow preprogrammed trajectories during the open-loop blocks and were specified by the decoding algorithm during the closed-loop blocks.

#### Open-loop finger task

Center-out-and-back trials were paired together. On the ‘center-out’ trials, one of the three finger groups was randomly chosen (or one of the two finger groups when training the 2D decoder) to move from the neutral position to either full flexion or full extension in 2 s and then hold for 1 s. The participant was asked to attempt movement of his fingers in sync with the virtual fingers following a smoothly varying trajectory. On the ‘back’ trial, the previously flexed or extended finger group would move back toward the neutral position and then hold for 1 s. Rest trials without finger movement were also included. All trials were included as training data for training decoder algorithms. As an illustration for comparison with previous and future finger classification work^18,19,26^, finger movements from one session, Session 6, were also classified using neural activity over long time windows typically used in classification (2 s) and short time windows typically used for closed-loop decoding (150 ms; Extended Data Fig. 1b,c).

#### Closed-loop 2D finger tasks

The closed-loop two-finger task was used for both training and testing the decoding algorithm. In this task, the participant controlled two simultaneous finger groups within a 1D arc: the thumb and index–middle group. On paired trials, the participant was cued to simultaneously move the finger groups from a center ‘neutral’ position toward random targets within the active range of motion. Once reaching the target, all fingers were required to be within the target for 500 ms for the trial to be successfully completed. On the subsequent trial, targets were placed back at the center. The target width was 20% of the range of motion, and the trial timeout time was 10 s.

#### Closed-loop 4D finger tasks

There were several 4D finger tasks used for training and testing the decoding algorithm. The most frequently tested 4D task, denoted 4T, allowed the participant to simultaneously control three finger groups: thumb with 2D movements of flexion/extension and abduction/adduction, the index–middle group with 1D movements of flexion/extension and the ring–little group with 1D movements in flexion/extension. In the first of paired trials, two new random targets would appear for two randomly selected finger groups, and the participant would be cued to move the fingers to the targets while keeping the third finger group stationary within its original central position target. The trial was completed successfully if all three finger groups were within their respective targets for 500 ms before a 10-s trial timeout. On the second of two paired trials, all targets would return to the center position, prompting the two moving fingers from the previous trial to return to center targets. A similar task (Extended Data Fig. 5a) had only one new target per trial. Finally, when training the quadcopter, a closed-loop random finger task was used, where two new random targets per trial appeared in the active range of motion for the finger groups; that is, there were no paired center-out-back trials, and each trial was independent of the previous.

The most-used task for training, denoted T_TRAIN_, was a four-DOF task similar to 4T above with several key differences so that intended decoder movements could be accurately inferred from a poorly/partially trained decoder. First, at the end of each trial, the positions of the fingers would return to the center position, which prevented fingers from becoming permanently stuck in flexion or extension. When two new targets were presented on a trial, the finger without a new target was artificially held fixed in the center position so that the participant could focus on only two finger groups per trial. The required hold time to successfully complete a trial was lengthened to 1.5 s to provide more training data when trying to steady the fingers, and trial timeout was reduced to 5 s so that the participant would not decrease his effort at the end of a longer trial. Finally, every other trial held the targets in the center position and the virtual fingers were fixed in place to provide an abundant amount of data where the participant was trying to remain stationary on the targets.

### Quadcopter tasks

To demonstrate the utility of closed-loop, online dexterous finger decoding in an applied task, finger control was mapped to 4D control of a virtual quadcopter. Specifically, the finger positions were mapped to a velocity-control paradigm, as shown in Fig. 4a. A physics-based quadcopter environment used the Microsoft AirSim plugin^42^ as a quadcopter simulator in Unity (v.2019.3.12f1). Two main tasks were developed to test this control: the quadcopter obstacle course that demonstrates control with all four DOF and the random ring-acquisition task in which the participant demonstrates spontaneous control using multiple DOF at the same time. The participant was given time to become comfortable with the control paradigm in some preliminary sessions and was then evaluated on the obstacle course and random ring task for 1 day each.

#### Quadcopter obstacle course

A virtual basketball court was created in Unity with two large rings placed along the long axis of the basketball court (Fig. 4b). To demonstrate control of all four DOF, a path through and around the rings was designed (Fig. 4b). During one day of testing, the participant was allowed unlimited trials to complete the obstacle with the goal of recording his personal best time, with instructions to complete all segments of the obstacle course as quickly and accurately as possible. He completed the course a total of 12 times, and during these completed runs, no penalty was assessed for not staying exactly on course, hitting rings or missing rings (he did miss two of the total 168 possible rings).

#### Random ring acquisition

On one day of testing, only one ring was displayed, which was randomly generated both in its location in space and orientation, and the participant navigated the quadcopter through these random rings. The rate of ring acquisition during the first 10 min was calculated, and a video of a representative time segment is included.

### Decoding algorithm

The decoding algorithm presented in ref. ^23^ was adapted for this work. The algorithm is a shallow-layer feed-forward neural network with an initial time-feature learning layer implemented as a scalar product of historical time bins and learned weights. A rectified linear unit was used as the nonlinearity after the convolutional layer and each linear layer except for the last linear layer. The input *Y*_IN_ was an *E*_N_ × 3 input matrix, where *E*_N_ is the number of electrodes (192) and 3 represents the three most recent 50-ms bins. The time-feature learning layer converts three 50-ms bins into 16 learned features using weights that are shared across all input channels. The output was flattened and then passed through four fully connected layers. The intermediate outputs were highly regularized with batch normalization (batchnorm)^43^ and 50% drop out. The output variable, ${\boldsymbol{\hat{{v}}}}$, represents an array of decoded finger velocities that, if ideally trained, would be normalized with zero mean with unit variance. However, an empirical mean value and standard deviation were subsequently calculated from the training dataset, which were used to normalize ${\boldsymbol{\hat{{v}}}}$, and then an empirically tuned gain was applied to the decoded finger velocities.

In a change from ref. ^23^, to reduce the ability of the neural network to produce velocities with non-zero means, the final linear layer was changed to disallow an affine output, and the final batchnorm layer was not allowed to learn a bias. Furthermore, during training and testing, the final batchnorm was not allowed to apply a mean correction, as only a variance correction was allowed. The purpose of these changes was to penalize the preceding algorithmic blocks during training if the decoded signal had a non-zero mean.

### Closed-loop decoding software

The SBP was imported to a script that calculated ${\boldsymbol{\hat{{v}}}}$ from the input data (three time bins, 192 channels). The signal ${\boldsymbol{\hat{{v}}}}$ was normalized using the values calculated during training, and the empirically tuned gain was also applied. No smoothing was applied. The positions of the fingers were updated at each time step using the velocities.

When the positions of the virtual fingers were used to control the quadcopter, ‘gravity’ was applied to the fingers when the fingers were near the neutral position so that the fingers did not appear to jitter when the intention was to hold them steady. Specifically, when the fingers were within 10% of the range of motion of the neutral position, a position-independent, constant, low-amplitude value was added to the decoded velocity of the finger to bias the velocity toward the neutral position. Decoded velocities were scaled to a maximum of ±10 m s^−1^ and ±90 deg s^−1^ for linear and rotational velocities, and each DOF was tuned empirically with gain values equal to 0.6 for thumb flexion/extension, 0.8 for thumb abduction/adduction, 0.4 for index–middle flexion/extension and 0.6 for ring–little flexion/extension.

### Algorithm training

The algorithm was trained on a combination of open- and closed-loop trials, and the details are included in the Supplementary Methods. Briefly, the algorithm (Extended Data Fig. 2) was initialized using the Kaiming initialization method^44^. The neural network minimized the mean-squared error (torch.nn.MSELoss) between the actual finger velocities during open-loop training and the algorithm output using the Adam optimization algorithm^45^ (torch.optim.Adam). After the offline algorithm training, the online, closed-loop sessions were performed. After a closed-loop session, the adapted recalibrated feedback intention-trained (ReFIT) algorithm^23,33^ was used to update the parameters of the neural network. The corresponding finger velocities used for training were assigned a value equal to the decoded velocity when the velocity is pointed toward the target, and the sign is inverted when the velocity is directed away from the target. Starting with the same parameters for the neural network algorithm used during the online session, the Adam optimization algorithm (lr = 1 × 10^−4^, weight_decay = 1 × 10^−2^) was applied and trained over 500 additional iterations.

### BCI rig and front-end signal processing

The BCI rig was set up in three distinct configurations as our lab transitioned from an older analog setup to the newer digital setup. The details are given in the Supplementary Methods.

### Training protocols for the 4D decoder

After the algorithm parameters were trained from the open-loop session, closed-loop control using T_TRAIN_, which was easier to control with a suboptimal decoder, was used until approximately 80% of trials were completed. Then the three-finger task, 4T, was used for 50 additional trials. After each closed-loop session, the algorithm parameters were updated according to the section (‘Algorithm training’).

As a control to understand how neural instabilities^46^ could affect decoding performance, the stability of the 4D decoder was evaluated during two research sessions by training an initial decoder, fixing the parameters and using this fixed decoder on consecutive blocks until trials could not be reliably completed. This occurred after 20 min (5 blocks) on the first day and 53 min (11 blocks) on the second. On the first day, the decoder was retrained to demonstrate recovery of performance with retraining (Extended Data Fig. 5c). Although not implemented here, several approaches could be explored to stabilize decoding to neural instabilities, including rapid decoder calibration^47^, training decoders using a long history of previously recorded data^48^, adaptive decoders using task knowledge^49,50^ and algorithms that perform dimensionality reduction to a stable manifold followed by realignment^51,52^.

On occasion the decoder was trained but the parameters required updating either to improve performance from an instability or for a fair comparison with another decoder. When this was required, a combination of T_TRAIN_ and 4T were used. The training of each decoder used in closed-loop sessions is described in Table 2.

### Training protocols for the 2D decoder

The 2D finger decoder was trained with open-loop sessions first and then with closed-loop sessions, like the 4D decoder. Unlike the 4D decoder, the two-finger task for the 2D decoder was the only task performed. Furthermore, on some occasions, the 2D decoder was trained until 100% of trials were completed successfully, and on other occasions training was continued even after 100% of trials were completed. The training of these decoders is also described in Table 2.

### Online metrics

The online metrics defined, including acquisition time, time to target, orbiting time, path length efficiency and throughput, were defined similar to previous reports^22^ and are detailed in the Supplementary Methods.

### Offline analyses

The offline analyses were conducted in Python (v.3.9.12) using a Jupyter notebook (https://jupyter.org/) and in MATLAB (v.2022a, Mathworks). The following Python packages were used: scipy (v.1.7.3), torch (v.1.12.0), torchvision (v.0.13.0), numpy (v.1.21.5), matplotlib (v.3.5.3), PIL (v.9.0.1) and sklearn (v.1.0.2). Confusion matrices and dimensionality analyses are detailed in the Supplementary Methods and are similar to analyses in previous reports^27,30^. Analysis of the 2D and 4D decoders on the 2D task primarily relies on the normalized CC function and is detailed in the Supplementary Methods.

#### Statistical analysis

All statistical comparisons used a two-sample, two-tailed *t*-test in MATLAB using the function ttest2.m. This function is used to report the *P* value, *t*-statistic, DOF and CIs.

#### dSNR

Although SNR metrics have been proposed for offline analyses, a vector-based SNR^53^ was adapted specifically for closed-loop decoding, denoted dSNR. In this formulation, ${{\mathbf{v}}}\left[n\right]={[{v}_{1}[n],{v}_{2}[n],\cdots {v}_{d}[n]]}^{T}$ is a normalized target vector, ||${{\mathbf{v}}}\left[n\right]$||$=1$, for *d* DOF with positive amplitudes for flexion/abduction and negative amplitudes for extension/abduction. Thus, in the 2D task, **v**[*n*], at a given 50-ms time bin, *n*, is represented graphically in Fig. 3a, where, as an example, ${{\mathbf{v}}}={[\mathrm{0.707,0.707}]}^{T}$ is a 2D vector indicating that both fingers require flexion to reach the target. The array of *d* decoded/predicted finger velocities, $\hat{{{{\mathbf{v}}}}}\left[n\right]={[{\hat{v}}_{1}[n],{\hat{v}}_{2}[n],\cdots {\hat{v}}_{d}[n]]}^{T}$, is assumed to be a time-varying, *d*-dimensional vector. This vector can be decomposed into orthogonal components, including a signal component, ${\boldsymbol{\hat{{v}}}}_{{{\bf{s}}}}\left[n\right]$, that is the projection of $\hat{{{\mathbf{v}}}}\left[n\right]$ along ${{\mathbf{v}}}\left[n\right]$, and a noise component, ${\boldsymbol{\hat{{v}}}}_{{{\bf{n}}}}\left[n\right]$, orthogonal to ${{\mathbf{v}}}\left[n\right]$, as graphically depicted in Fig. 3a for the 2D task. Using this formulation, dSNR is defined in equation 2:

2 $${\rm{dSNR}}=E\left[|\left|{\boldsymbol{\hat{{v}}}}_{\bf{s}}\right||\right]\left/\sqrt{E\left[{{||}{\boldsymbol{\hat{{v}}}}_{\bf{n}}{||}}^{2}\right]}\right.$$

The value of dSNR was empirically calculated from closed-loop blocks of two and three decoded fingers (Table 2) during the ‘Go’ period of the trials (200–700 ms after a new target was presented) before fingers were on their respective targets. To empirically calculate dSNR, the SBP data are divided into six folds: five training folds and one testing fold. To regularize the number of regressors (that is, 192 channels) for linear regression, principal component analysis decomposition was used (sklearn.decomposition.PCA) on the *n* 50-ms time bins by *E*_N_ = 192 input channels (*n* × 192) of SBP training data, *X*_TRAIN_, to reduce the number of dimensions to an *n* × 20 dataset, ${\widetilde{{{X}}}}_{{\rm{TRAIN}}}$. Using LinearRegression from sklearn.linear_model toolbox, a linear mapping is trained to map ${\widetilde{{{X}}}}_{{\rm{TRAIN}}}$ to the *n* × *d* training velocities, *V*_TRAIN_ (that is, **v** in Fig. 3a). These commands are represented with the pseudocode in equations (3)–(6):

3 $${\rm{pca}}={\rm{PCA}}({\rm{n\_components}}=20)$$

4 $${\rm{pca}}.{\rm{fit}}({{{X}}}_{{\rm{TRAIN}}})$$

5 $${\widetilde{{{X}}}}_{{\rm{TRAIN}}}={\rm{pca}}.{\rm{transform}}({{{X}}}_{{\rm{TRAIN}}})$$

6 $${\rm{reg}}1={\rm{LinearRegression}}().{\rm{fit}}({{{X}}}_{{\rm{TRAIN}}},\,{{{{\it{V}}}}}_{{\rm{TRAIN}}})$$

Finally, the predicted velocities, ${\widehat{{{{\it{V}}}}}}_{{\rm{TEST}}}$, of the test data, ${\widetilde{{{X}}}}_{{\rm{TEST}}}$, were determined from equation (7):

7 $${\hat{{{{\it{V}}}}}}_{{\rm{TEST}}}={\rm{reg}}1.{\rm{predict}}({\widetilde{{{X}}}}_{{\rm{TEST}}})$$

The predicted finger velocities, ${\widehat{{{{\it{V}}}}}}_{{\rm{TEST}}}$, for the 2D decoder are shown in Fig. 3b. The magnitude of the signal component of the predicted velocity, ${||}{\boldsymbol{\hat{{v}}}}_{\bf{s}}{||}$ as in Fig. 3a, was calculated from the dot product of ${\widehat{{{\it{V}}}}}_{{\rm{TEST}}}$ and ${{{\it{V}}}}_{{\rm{TEST}}}$ according to equation (8):

8 $${||}{\boldsymbol{\hat{{v}}}}_{\bf{s}}{||}={\rm{np}}.{\rm{sum}}({\widehat{{{\it{V}}}}}_{{\rm{TEST}}}* {{{\it{V}}}}_{{\rm{TEST}}},{\rm{axis}}=1)$$

where * denotes element-by-element multiplication, ${||}{\boldsymbol{\hat{{v}}}}_{\bf{s}}{||}$ is a length-*n* array for *n* time steps. To compute the noise component, $\left|\left|{\boldsymbol{\hat{{v}}}}_{\bf{n}}\right|\right|$, *θ*, the angle between ${\boldsymbol{\hat{{v}}}}_{\bf{s}}$ and **v** in Fig. 3a and ${||}{\boldsymbol{\hat{{v}}}}_{\bf{n}}{||}$ were calculated according to equations (9) and (10). Finally, in equation (11), the value of dSNR was calculated:

9 $$\theta ={\rm{np}}.\arccos ({{||}}{\boldsymbol{\hat{{v}}}}_{\bf{s}}{{||}}\big/{\rm{np}}.{\rm{sqrt}}({\rm{np}}.{\rm{sum}}({\widehat{{{\it{V}}}}}_{{\rm{TEST}}}{**} 2,{\rm{axis}}=1)))$$

10 $$\left|\left|{\boldsymbol{\hat{{v}}}}_{\bf{n}}\right|\right|={\rm{np}}.\sin ({{\theta }})* {\rm{np}}.{\rm{sqrt}}({\rm{np}}.{\rm{sum}}({\widehat{{{\it{V}}}}}_{{\rm{TEST}}}{**} 2,{axis}=1))$$

11 $${\rm{dSNR}}={\rm{np}}.{\rm{mean}}({{||}}{\boldsymbol{\hat{{v}}}}_{\bf{s}}{{||}})\left/{\rm{np}}.{\rm{sqrt}}({\rm{np}}.\mathrm{var}(||{\boldsymbol{\hat{{v}}}}_{\bf{n}}||)+{\rm{np}}.{\rm{mean}}(||{\boldsymbol{\hat{{v}}}}_{\bf{n}}||){**} 2)\right.$$

where ** denotes an exponent. The value of dSNR was then averaged over all six folds. The data in ${\widehat{{{\it{V}}}}}_{{\rm{TEST}}}$ for all folds and all days are the population data, shown for the 2D decoder in Fig. 3b.

To calculate dSNR as a function of channel count, dSNR was calculated for an array of input channels, *N*_C_[*k*], indexed by *k* and ranging from 5 to the full *E*_N_ = 192 at a step size of *E*_N_/20. At each step, the value of dSNR was averaged over 25 iterations, where at each iteration, *N*_C_[*k*] random input channels were selected.

The empirical fit for the log of dSNR averaged over all days and log of *N*_C_ was calculated using data from the highest 75% of values of *N*_C_ and using numpy.linalg.lstsq for the empirical fit and sklearn.metrics.r2_score for the coefficient of determination, *R*^2^. A theoretical derivation for the dependency of dSNR on channel count is given in the Supplementary Methods.

### Reporting summary

Further information on research design is available in the Nature Portfolio Reporting Summary linked to this article.

## Online content

Any methods, additional references, Nature Portfolio reporting summaries, source data, extended data, supplementary information, acknowledgements, peer review information; details of author contributions and competing interests; and statements of data and code availability are available at 10.1038/s41591-024-03341-8.

## Supplementary information

Supplementary Information Supplementary Methods

Reporting Summary

Supplementary Video 1 The 2D decoder on the 2D task with a mean acquisition time of 0.84 ± 0.05 s, corresponding to a target acquisition rate of 143 targets per minute.

Supplementary Video 2 The 4D decoder on the 4D task with two new targets per trial, with a mean acquisition time of 1.48 ± 0.09 s, corresponding to a target acquisition rate of 81 targets per minute.

Supplementary Video 3 The 4D decoder on the 4D task with one new target per trial trial, with a mean acquisition time of 1.08 ± 0.09 s, corresponding to a target acquisition rate of 56 targets per minute.

Supplementary Video 4 The 4D decoder on the 2D task when two DOF are fixed and not allowed to move (thumb abduction/adduction and ring–little flexion/extension). The mean acquisition time was 1.07 ± 0.07 s, corresponding to a target acquisition rate of 112 targets per minute.

Supplementary Video 5 Exemplar block of the finger iBCI translated to control a quadcopter with four DOF during an obstacle course presented in Fig. 4b and flight path shown in Fig. 4c.

Supplementary Video 6 Using the finger iBCI translated to quadcopter control to navigate through randomly appearing rings to demonstrate spontaneous, free-form control.

## Figures and Tables

**Extended Data Table 1:**
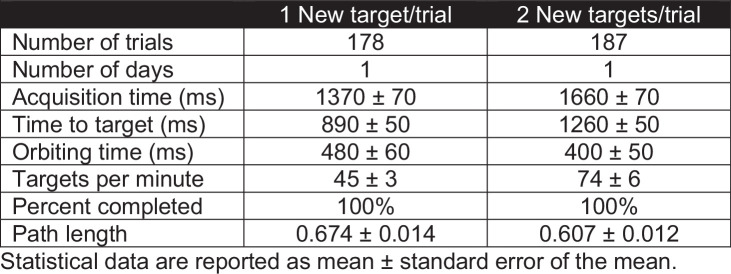
Performance metrics for 4D finger decoding with 1 and 2 new targets per trial

**Extended Data Table 2:**
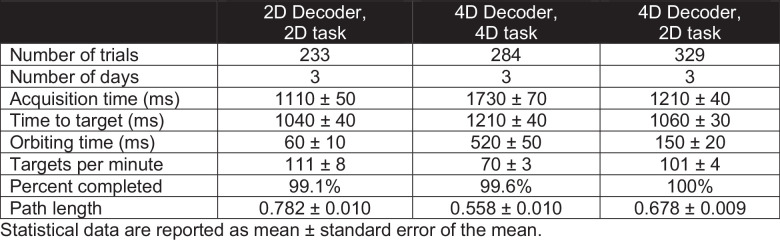
Performance metrics for finger decoding using 4D decoder on 2D task

## Data Availability

Data needed to reproduce the key findings in this study are publicly available on Dryad at 10.5061/dryad.1jwstqk4f (ref. ^54^).

## Code availability

Code to implement the offline analysis of the central findings of this study is publicly available on GitHub at https://github.com/WillseyBCILab/BCI_Finger_Decoding_Quadcopter.
