# Supplementary material for: A high-performance brain–computer interface for finger decoding and quadcopter game control in an individual with paralysis
Source: Nat Med. 2025 Jan 20;31(1):96–104. doi: 10.1038/s41591-024-03341-8 (PMC11750708; doi:10.1038/s41591-024-03341-8)
Supplement: Supplementary file 1 — Supplementary Methods [file 41591_2024_3341_MOESM1_ESM.pdf]

nature medicine

Supplementary information https://doi.org/10.1038/s41591-024-03341-8

A high-performance brain–computer interface for finger decoding and quadcopter game control in an individual with paralysis

In the format provided by the authors and unedited

Supplementary Methods

Offline algorithm training

The algorithm was trained on a combination of open- and closed-loop trials. For each day, the algorithm was trained on 2 blocks of 100 open-loop trials. The SBP data were organized into batches of 64x256x3 (64 randomly selected time steps, 256 input channels, 3 previous time bins adjacent in time to the current time step). The velocities of the virtual fingers during the open-loop block were normalized by the standard deviation of the velocity and then multiplied by 0.2 (referred to as `stretchFactor`), as a reduced amplitude of open-loop finger velocities was previously observed to yield a better offline fit[1]. Thus, the finger velocities used for training were (in pseudocode):

$\mathtt{Y_{Train}\ =\ 0.2*Y_{RAW}/torch.std(Y_{RAW},axis=0)}$ Eq. 1

where $\mathrm{Y_{RAW}}$ was an array of d finger velocities for N training samples ($N \times d$).

The algorithm (Extended Data Fig. 2) was initialized using the Kaiming initialization method[2]. The neural network minimized the mean-squared error (torch.nn.MSELoss) between the actual finger velocities during open-loop training and the algorithm output using Adam optimization algorithm[3] (torch.optim.Adam). The optimizer was used with a learning rate of 10^{-4} and weight decay of 10^{-2} (parameters: lr=1e-4, weight_decay=1e-2), and the algorithm was trained over 10 epochs.

After training the algorithm, a mean offset value, μ_{offset}, was calculated (Eqs. 3 and 4) to give the output of the neural network algorithm the same mean as the unnormalized training data.

$\mathtt{\mathbf{v}_{hat}\ =\ model.forward(\mathbf{X}_{TRAIN})}$ Eq. 2

$\mathtt{\boldsymbol{\mu}_{offset}=\ np.mean(\mathbf{v}_{hat},axis=0)\ -\ np.mean(\mathbf{Y}_{RAW},axis=0)}$ Eq. 3

The variable $\mathbf{v}_{hat}$ is the $(N \times d)$ output tensor after applying the trained neural network algorithm (`model`), $\mathbf{X}_{TRAIN}$ is a $N \times E_N \times 3$ tensor array for the N training time steps, E_N input channels, and 3 preceding time steps to the current time step. The variable $\mathbf{v}_{hat}$ was converted to a numpy array and input to Eq. 2, and Y_{RAW} was defined in Eq. 1. A gain for the algorithm output, G, was calculated to normalize the standard deviation of the algorithm output and further reduce the output amplitude by a factor of 3 (which was empirically determined):

```
G = 1/(3*np.std(vhat,0))
```
Eq. 4

Thus, when running the algorithm for online, closed-loop decoding, the output for each time step was adjusted according to Eq. 5:

$$P_F[n] = P_F[n-1] + 0.05 \cdot G \cdot \left(f_{Model}(X[n]) - \mu_{offset}\right) \quad \text{Eq. 5}$$

where $P_F[n]$ denotes the position of d finger groups at time step n.

After the offline algorithm training, the online, closed-loop sessions were performed. After a closed-loop session, the adapted recalibrated feedback intention-trained (ReFIT) algorithm[1,4] was used to update the parameters of the neural network. Similar to above, SBP data were organized into 64x256x3 batches, with the 64 time steps randomly selected. The corresponding finger velocities used for training were assigned a value equal to the decoded velocity when the velocity is pointed toward the target, and the sign is inverted when the velocity is directed away from the target (Eq. 6):

```
YREFIT = torch.sign(PT - PF)*torch.sign(YRAW)*YRAW
```
Eq. 6

where P_T is the position of the target, P_F is the position of the fingers, and Y_{RAW} was an array of d finger velocities for N training samples $(N \times d)$. Similar to offline training, the velocities were then scaled by the standard deviation and a `stretchFactor` of 1.3 (promoting higher velocities toward the target), except when the finger positions lie within the target when

`stretchFactor` divides the value of Y_{REFIT} (promoting lower velocities). These steps were implemented by executing the 2 consecutive lines of pseudocode given in Eqs. 8-9:

```
YTrain = 1.3*YRAW/torch.std(YREFIT,axis=0)                          Eq. 7
YTrain[PT-PF < TS/2] = YTrain[PT-PF < TS/2]/1.3/1.3                 Eq. 8
```

Of note, dividing twice by `1.3` in Eq. 8 is required to undo the `1.3` multiplication factor in Eq. 7. Starting with the same parameters for the neural network algorithm used during the online session, the Adam optimization algorithm (lr=1e-4, weight_decay=1e-2) was applied and trained over 500 additional iterations. A new value for μ_{offset} was calculated according to Eq. 7 using `np.median` instead of `np.mean`, and *G* was calculated as before in Eq. 4.

```
μoffset= np.median(vhat,0) - np.median(YREFIT,0)                    Eq. 9
```

When running the algorithm online, the finger positions were again updated according to Eq. 5.

BCI rig and front-end signal processing

The BCI rig was set up in 3 distinct configurations as our lab transitioned from an older analog setup to the newer digital setup. In the first setup used in sessions 1-7, 2 patient cables were connected to the transcutaneous pedestals, which were routed to the Neural Signal Front End Amplifier (Blackrock Neurotech, Salt Lake City, Utah) where the raw voltage was bandpass filtered (0.3 Hz first-order high-pass and 7.5 kHz third-order low-pass), sampled at 30 kHz with 250 nV resolution, converted to an optical signal, and then sent to the Neural Signal Processor[5]. In session 9, 2 Neuroplex E headstages were connected to 2 transcutaneous pedestals, and the signal was analog filtered and sampled at the headstage and then sent to the digital hub via a micro-HDMI cable. At the digital hub, the signal was converted to an optical signal transmitted via optical cable to the Neural Signal Processor. In the final mixed configuration, used in session

8, the setup using the analog patient cable was connected to the anterior pedestal, and a Neuroplex E headstage with its subsequent configuration was connected to the posterior pedestal.

The Neural Signal Processor sends the digital signal to a SuperLogics machine running Simulink Real-Time (v2019, Mathworks, Natick, MA). For most sessions, common average referencing (CAR) was used to reduce the electrical noise on the input channels. However, during both quadcopter sessions (sessions 8 and 9), CAR was switched to linear regression referencing to predict a reference from the combined input channels that is subtracted from each input channel[6]. The signals then passed through a 250-Hz digital high-pass filter. The data were binned into 50-ms windows. The sum of the squared magnitude was calculated for each window. This signal was denoted as spike-band power (SBP). Every 50 ms, UDP packets of neural features are communicated to a Linux computer running Ubuntu with Python (v3.7.11), PyTorch (v1.12.1, https://pytorch.org/), and Redis (v7.02), where the neural features pass into the decoding algorithm. The entire system was interfaced with an additional Windows computer running Matlab (v2019, Mathworks, Natick, MA) that was interfaced with the system to stop and start experimental blocks during sessions.

Online performance metrics

Various metrics were calculated to characterize online performance. Trials in which fingers started within a "new" target were excluded from the analysis. Performance metrics were only calculated from successful trials. The acquisition time was defined as the time from the start of the trial to when the fingers had successfully held on each of the targets for the required hold time of 500 ms subtracted by 500 ms. The time to target was defined as the time from the start of

the trial to when all fingers reached the target (and did not require all fingers to be on the target simultaneously). The orbiting time was the acquisition time subtracted by the time to target. Targets per minute was the number of new targets per trial divided by the mean acquisition time (excluding hold time). Successfully completing the tasks required completing the task within 10 s (i.e., acquisition time of 9.5 s). For completeness, the path length efficiency was calculated, although a metric perhaps more suitable for single-effector *d*-dimension control. Path length for each trial was calculated according to Eq. 10:

$$L_P = \frac{\| \|\boldsymbol{P_F}[N] - \boldsymbol{P_F}[0]\| \|}{\sum_{k=1}^{N} \|\boldsymbol{P_F}[k] - \boldsymbol{P_F}[k-1]\|} \qquad \text{Eq. 10}$$

where ||·|| denotes the L_2 norm, L_p is the path length efficiency for that trial, N+1 is the elapsed samples until all fingers are on the target, and $\boldsymbol{P_F}$ is a vector of the positions of all d fingers. Thus, L_p close to unity implies the fingers traveled a direct path to the targets, and a value close to zero implies a circuitous path to the target. Finally, when calculating the throughput in bits per second (bps), T_{bps}, the same adaption of Fitt's law for fingers developed in Willsey et al.[1] was used and is repeated in Eq. 11:

$$T_{bps} = \frac{log_2(3) + \sum_k log_2\left(1 + \frac{(D_k - S)}{2S}\right)}{t_{acq}} \qquad \text{Eq. 11}$$

where k indexes through all 3 finger groups, Dk is the distance of the k-th to the target, S is the circular target radius, and t_{acq} is the target acquisition time. T_{bps} was then averaged over all trials. For this calculation, the only trials included are those for which all fingers begin a distance from the center of the target that is greater than twice the target radius. In an adaption to the approach in Willsey et al.,[1] $\log_2(3)$ bits was added on each trial to account for the information needed to convey which finger is stationary. An alternative approach would be to not add these additional

bits and allow the T_{bps} to decrease with the understanding that the total corpus of targets is higher.

To illustrate how decoded finger movements could be discriminated, the mean decoded velocity was calculated during single-finger movements. This analysis is shown in Fig. 1f. Four blocks of the 4D task with 1 new target/tr (Extended Data Fig. 5a) were used for this analysis. On each trial, the mean velocity of all fingers was calculated during the 'Go' period (200-700 ms after trial start) and normalized by the mean value of the finger group with the highest mean value (which was the cued finger).

Confusion matrices

The fingers were classified during open-loop trials to relate the tuning of these arrays to other reports focusing on classification[7,8,9]. Classification over a 2-s movement window was used to illustrate performance over typical windows used for classification and over a shorter 150-ms window similar to windows used for closed-loop decoding. The open-loop data from a typical day, session 6, were used for this analysis (200 trials and 192 input channels of SBP). Both a 10-fold cross-validation and a linear discriminant analysis classifier that assumes a shared diagonal covariance matrix across conditions were used. The analysis was performed in Matlab 2022a using the functions: fitcdiscr.m (with 'DiscrimType' as 'diaglinear'), crossval.m, kfoldLoss.m, and kfoldPredict.m.

Dimensionality

While there are numerous approaches to calculate dimensionality[10], the participation ratio was used, which is roughly equivalent to the dimensions needed to capture 80% of the

variance[11,12]. The 'Go' period during the trial, 200-700 ms after a new target appeared, was averaged for each condition. A $d \times 1$ condition vector, $\boldsymbol{C}_{DOF}$, was defined according to whether each respective DOF at the beginning of the trial needed to flex/abduct (+1), extend/adduct (-1), or remain stationary (0) to reach the targets. Thus, for 4D closed-loop decoding during the 4D finger task, $\boldsymbol{C}_{DOF} = [1,1,0,-1]^T$ if the thumb needed to flex and abduct to reach the target, the index-middle group needed to remain on the target, and the ring-little group needed to extend. The SBP for each electrode during the 'Go' period was z-scored and smoothed using scipy.ndimage.gaussian_filter1d with a sigma of 3 50-ms time bins. The data were then organized into a matrix, $\boldsymbol{D}_{4d}$, that was $E_N \times (cN_M)$, where E_N is the integer 192 for the number of input channels, c is the integer 20 for the number of conditions in the 4D task with 2 new targets/trial, and N_M is the integer 10 for the number of 50-ms bins in the 'Go' period. Similar data matrices were calculated for the 2D task, $\boldsymbol{D}_{2d}$, and for the 4D task with 1 target/trial, $\boldsymbol{D}_{4d1t}$. The eigenvalues, u_i, were then calculated for the cross-validated covariance matrix, $\boldsymbol{D}_p\boldsymbol{D}_q^T$ (where $\boldsymbol{D}_p$ and $\boldsymbol{D}_q$ were data matrices from 2 folds of the data). The participation ratio was calculated (Eq. 12).

$$PR = \frac{(\sum_i U_i)^2}{\sum_i {U_i}^2} \quad \text{Eq. 12}$$

Analysis of the 2D and 4D decoders on the 2D task

To determine whether mapping changes when mapping neural activity to a 4D vs 2D task, decoders were trained on the 4D and 2D tasks as explained above and both of these decoders were used for the 2D task (thumb flexion/extension and index-middle flexion/extension). To compare these mappings, the 2D decoding algorithm was used to predict the velocities when using the 4D decoder in closed-loop trials, and the 4D algorithm was used to

predict the closed-loop decoded velocities of the 2D algorithm. Fig. 2c illustrates velocities decoded online by the 2D decoder and predicted by the 4D decoder. To quantify the similarity between these signals, the normalized cross correlation, r_n, function was calculated as defined below in Eq. 13,

$$r_n = \frac{\sum_n \hat{v}_D[n] \cdot \hat{v}_P[n]}{\sqrt{\sum_n \hat{v}_D[n] \cdot \hat{v}_D[n]}\sqrt{\sum_n \hat{v}_P[n] \cdot \hat{v}_P[n]}} \qquad \text{Eq. 13}$$

where $\hat{v}_D$ is the velocity decoded during the online block and $\hat{v}_P$ is the velocity predicted offline. The value for r_n was then averaged for both DOF. For paired blocks with the 4D and 2D decoders, the normalized cross correlation function was calculated, and these data are displayed in Fig. 2d.

Theoretical SNR dependency on channel count

For a theoretical comparison for the dependency of dSNR on channel count, a 1D signal, S, measured independently on N channels was defined according to the form:

$$\boldsymbol{S} = \frac{1}{N}\sum_N (s_k + \eta_k) \qquad \text{Eq. 14}$$

where s_k is the signal and η_k is i.i.d. Gaussian samples from the distribution $N(0, \sigma)$. Assuming for simplicity and without loss of generality that s_k are equal, then Eq. 14 simplifies to:

$$\boldsymbol{S} = s + \frac{1}{N}\sum_N \eta_k \qquad \text{Eq. 15}$$

Thus, the value of the expected signal in Eq. 14 equals simply s. The expected square of the noise, $E[\boldsymbol{\eta}^2]$, can be simplified to:

$$E[\boldsymbol{\eta}^2] = E\left[\left(\frac{1}{N}\sum_N \eta_k\right)^2\right] = \frac{1}{N^2}E[(\sum_N \eta_k)^2] = \frac{1}{N^2}\sum_N E[{\eta_k}^2] \qquad \text{Eq. 16}$$

where the last equality follows since the terms η_k are independent. Finally,

$$E[\boldsymbol{\eta}^2] = \frac{1}{N^2}\sum_N E[{\eta_k}^2] = \frac{1}{N^2}N\sigma^2 = \frac{\sigma^2}{N}. \qquad \text{Eq. 17}$$

Thus, the SNR in this simplified case is:

$$SNR = {}^{E[\boldsymbol{S}]}\Big/_{\sqrt{E[\boldsymbol{\eta}^2]}} = \sqrt{N} \cdot {}^{s}/_{\sigma}. \qquad \text{Eq. 18}$$

Thus, for our definition of SNR as defined in Eq. 2 of the Methods, the SNR increases proportionally with $\sqrt{N}$ in this theoretical, simplified 1D formulation.

Supplementary Methods References

1 Willsey, M. S. *et al.* Real-time brain-machine interface in non-human primates achieves high-velocity prosthetic finger movements using a shallow feedforward neural network decoder. *Nat Commun* **13**, 6899 (2022).

2 He, K., Zhang, X., Ren, S. & Sun, J. Delving deep into rectifiers: Surpassing human-level performance on imagenet classification, in *Proceedings of the IEEE international conference on computer vision*. 1026-1034.

3 Kingma, D. P. & Ba, J. Adam: A method for stochastic optimization. *arXiv preprint arXiv:1412.6980* (2014).

4 Gilja, V. *et al.* A high-performance neural prosthesis enabled by control algorithm design. *Nat Neurosci* **15**, 1752 (2012).

5 Blackrock Microsystems. NeuroPort Biopotential Signal Processing System: Instructions for Use. (2023).

6 Young, D. *et al.* Signal processing methods for reducing artifacts in microelectrode brain recordings caused by functional electrical stimulation. *J Neural Eng* **15**, 026014 (2018).

7 Guan, C. *et al.* Decoding and geometry of ten finger movements in human posterior parietal cortex and motor cortex. *J Neural Eng* **20**, 036020 (2023).

8 Jorge, A., Royston, D. A., Tyler-Kabara, E. C., Boninger, M. L. & Collinger, J. L. Classification of individual finger movements using intracortical recordings in human motor cortex. *Neurosurgery* **87**, 630-638 (2020).

9 Shah, N. P. *et al.* Pseudo-linear summation explains neural geometry of multi-finger movements in human premotor cortex. *bioRxiv*, 2023.2010. 2011.561982 (2023).

10 Camastra, F. & Staiano, A. Intrinsic dimension estimation: Advances and open problems. *Information Sciences* **328**, 26-41 (2016).

11 Willett, F. R., Avansino, D. T., Hochberg, L. R., Henderson, J. M. & Shenoy, K. V. High-performance brain-to-text communication via handwriting. *Nature* **593**, 249-254 (2021).

12 Gao, P. *et al.* A theory of multineuronal dimensionality, dynamics and measurement. *BioRxiv*, 214262 (2017).
