# Supplementary material for: A high-performance brain–computer interface for finger decoding and quadcopter game control in an individual with paralysis
Source: Nat Med. 2025 Jan 20;31(1):96–104. doi: 10.1038/s41591-024-03341-8 (PMC11750708; doi:10.1038/s41591-024-03341-8)
Supplement: Supplementary file 2 — Reporting Summary [file 41591_2024_3341_MOESM2_ESM.pdf]

nature portfolio

Corresponding author(s): Matthew Willsey
Jaimie Henderson

Last updated by author(s): Sep 20, 2024

Reporting Summary

Nature Portfolio wishes to improve the reproducibility of the work that we publish. This form provides structure for consistency and transparency in reporting. For further information on Nature Portfolio policies, see our Editorial Policies and the Editorial Policy Checklist.

Statistics

For all statistical analyses, confirm that the following items are present in the figure legend, table legend, main text, or Methods section.

n/a	Confirmed	
☐	☒	The exact sample size (*n*) for each experimental group/condition, given as a discrete number and unit of measurement
☒	☐	A statement on whether measurements were taken from distinct samples or whether the same sample was measured repeatedly
☐	☒	The statistical test(s) used AND whether they are one- or two-sided<br>*Only common tests should be described solely by name; describe more complex techniques in the Methods section.*
☒	☐	A description of all covariates tested
☒	☐	A description of any assumptions or corrections, such as tests of normality and adjustment for multiple comparisons
☐	☒	A full description of the statistical parameters including central tendency (e.g. means) or other basic estimates (e.g. regression coefficient) AND variation (e.g. standard deviation) or associated estimates of uncertainty (e.g. confidence intervals)
☐	☒	For null hypothesis testing, the test statistic (e.g. *F*, *t*, *r*) with confidence intervals, effect sizes, degrees of freedom and *P* value noted<br>*Give P values as exact values whenever suitable.*
☒	☐	For Bayesian analysis, information on the choice of priors and Markov chain Monte Carlo settings
☒	☐	For hierarchical and complex designs, identification of the appropriate level for tests and full reporting of outcomes
☒	☐	Estimates of effect sizes (e.g. Cohen's *d*, Pearson's *r*), indicating how they were calculated

Our web collection on statistics for biologists contains articles on many of the points above.

Software and code

Policy information about availability of computer code

Data collection	The virtual finger display was developed in Unity (2021.3.9f1). A physics-based quadcopter environment used the Microsoft AirSim plugin41 as a quadcopter simulator in Unity (2019.3.12f1). The Neural Signal Processor sends the digital signal to a SuperLogics machine running Simulink Real-Time (v2019, Mathworks, Natick, MA). This computer communicated with a Linux computer running Ubuntu with Python (v3.7.11), PyTorch (v1.12.1, https://pytorch.org/), and Redis (v7.02), where custom code performed decoding, control of the virtual environment in Unity, and training of the neural network. The entire system was interfaced with an additional Windows computer running Matlab (v2019, Mathworks, Natick, MA) that was interfaced with the system to stop and start experimental runs during sessions.
Data analysis	The offline analyses were conducted in Python (v3.9.12) using a Jupyter notebook (https://jupyter.org/) and in Matlab (v2022a, Mathworks, Natick, MA). The following python packages were used: scipy (v1.7.3), torch (v1.12.0), torchvision (v0.13.0), numpy (v1.21.5), matplotlib (v3.5.3), PIL (v9.0.1), sklearn (v1.0.2). The versions of the Jupyter core packages are: IPython 8.2.0, ipykernel 6.9.1, ipywidgets 7.6.5, jupyter_client 6.1.12, jupyter_core 4.9.2, jupyter_server 1.13.5, jupyterlab 3.3.2, nbclient 0.5.13, nbconvert 6.4.4, nbformat 5.3.0, notebook 6.4.8, qtconsole 5.3.0, traitlets 5.1.1.<br><br>The Code Availability section now reads: Code to implement the offline analysis of the central findings of this study is publicly available on GitHub at https://github.com/WillseyBCILab/BCI_Finger_Decoding_Quadcopter.

For manuscripts utilizing custom algorithms or software that are central to the research but not yet described in published literature, software must be made available to editors and reviewers. We strongly encourage code deposition in a community repository (e.g. GitHub). See the Nature Portfolio guidelines for submitting code & software for further information.

Data

Policy information about availability of data

All manuscripts must include a data availability statement. This statement should provide the following information, where applicable:

- Accession codes, unique identifiers, or web links for publicly available datasets
- A description of any restrictions on data availability
- For clinical datasets or third party data, please ensure that the statement adheres to our policy

Data needed to reproduce the key findings in this study are publicly available on Dryad at https://doi.org/10.5061/dryad.1jwstqk4f.

Research involving human participants, their data, or biological material

Policy information about studies with human participants or human data. See also policy information about sex, gender (identity/presentation), and sexual orientation and race, ethnicity and racism.

Reporting on sex and gender	This study included data from one participant, T5, who is a biological male. No sex or gender based analyses were performed given there was only a single participant and the study was assessing brain-computer interface performance.
Reporting on race, ethnicity, or other socially relevant groupings	There was no reporting on race, ethnicity, or other socially relevant groupings.
Population characteristics	This study includes data from one participant (identified as T5) who gave informed consent and was enrolled in the BrainGate2 Neural Interface System clinical trial (CliniclaTrials.gov Identifier: NCT00912041, registered June 3, 2009) but this study did not report clinical trial results. T5 is a right-handed man, 69 years old during data collection with with C4 AIS C spinal cord injury.
Recruitment	Participant T5 was enrolled in the BrainGate 2 clinical trial after meeting inclusion criteria based in part on disease characteristics. Inclusion and exclusion criteria are available online (ClinicalTrials.gov). This investigation was pursued as part of the secondary outcome measure of the clinical trial. Regarding potential selection biases, the strict inclusion and exclusion criteria could lead to selecting healthier participants than the typical population with paralysis.
Ethics oversight	The BrainGate2 Neural Interface System clinical trial was approved under an Investigation Device Exemption (IDE) by the US Food and Drug Administration (IDE #G09003). Permission was also granted by the Institutional Review Board of Stanford University (protocol #52060) and the Mass General Brigham IRB (protocol #2009P000505). All research was performed in accordance with relevant guidelines/regulations.

Note that full information on the approval of the study protocol must also be provided in the manuscript.

Field-specific reporting

Please select the one below that is the best fit for your research. If you are not sure, read the appropriate sections before making your selection.

☒ Life sciences ☐ Behavioural & social sciences ☐ Ecological, evolutionary & environmental sciences

For a reference copy of the document with all sections, see nature.com/documents/nr-reporting-summary-flat.pdf

Life sciences study design

All studies must disclose on these points even when the disclosure is negative.

Sample size	No sample-size calculation was performed. Data were collected in a single participant to characterize the performance of a brain-computer interface. The number of trials are comparable to similar studies, and uncertainty in performance estimates were quantified using the standard error of the mean.
Data exclusions	No data was excluded. Finger decoding sessions were performed over a series of days (from March to April, 2023). The 4DOF obstacle course and random ring task were demonstrated on a single day after preliminary sessions to develop each task.
Replication	Primary results of finger decoding were evaluated successfully confirmed on 7 independent days without exclusion.
Randomization	There was not randomization of participants to treatment groups as there was only one participant. There was randomization of the positions of center-out-center finger targets to randomly sample the potential target space.
Blinding	There was no blinding related to treatment groups as there was only one participant enrolled. It was not possible to blind the participant to the task he was performing.

Reporting for specific materials, systems and methods

We require information from authors about some types of materials, experimental systems and methods used in many studies. Here, indicate whether each material, system or method listed is relevant to your study. If you are not sure if a list item applies to your research, read the appropriate section before selecting a response.

Materials & experimental systems

n/a	Involved in the study	
☒	☐	Antibodies
☒	☐	Eukaryotic cell lines
☒	☐	Palaeontology and archaeology
☒	☐	Animals and other organisms
☒	☐	Clinical data
☒	☐	Dual use research of concern
☒	☐	Plants

Methods

n/a	Involved in the study	
☒	☐	ChIP-seq
☒	☐	Flow cytometry
☒	☐	MRI-based neuroimaging

Plants

Seed stocks	n/a
Novel plant genotypes	n/a
Authentication	n/a
